# Prevalence of Physical Activity among Adolescents from 105 Low, Middle, and High-Income Countries

**DOI:** 10.3390/ijerph17093145

**Published:** 2020-04-30

**Authors:** Adilson Marques, Duarte Henriques-Neto, Miguel Peralta, João Martins, Yolanda Demetriou, Dorothea M. I. Schönbach, Margarida Gaspar de Matos

**Affiliations:** 1 Centro Interdisciplinar de Estudo da Performance Humana, Faculdade de Motricidade Humana, Universidade de Lisboa, 1499-002 Lisboa, Portugal; duarteneto@campus.ul.pt (D.H.-N.); mperalta@fmh.ulisboa.pt (M.P.); jmartins@fmh.ulisboa.pt (J.M.); 2 Instituto de Saúde Ambiental, Universidade de Lisboa, 1649-028 Lisboa, Portugal; mmatos@fmh.ulisboa.pt; 3 Laboratório de Pedagogia, Faculdade de Motricidade Humana e UIDEF, Instituto de Educação, Universidade de Lisboa, 1649-013 Lisboa, Portugal; 4 Department of Sport and Health Sciences, Technical University of Munich, 80333 Munich, Germany; yolanda.demetriou@tum.de (Y.D.); dorothea.schoenbach@tum.de (D.M.I.S.); 5 Faculdade de Motricidade Humana, Universidade de Lisboa, 1499-002 Lisboa, Portugal

**Keywords:** sports, physical exercise, young people, physical inactivity

## Abstract

Introduction: Physical activity (PA) is a beneficial health behaviour, however most adolescents worldwide are physically inactive. Updated information on the prevalence and trends of PA is important to inform national and international authorities and support countries’ public health policies and actions. This study aimed to present the worldwide, regional, and national prevalence of PA participation according to its frequency in adolescents. Methods: This study is based on cross-sectional surveys of adolescents’ populations from several countries and all regions worldwide. The sample comprised 520,533 adolescents (251,788 boys; 268,745 girls), from 105 countries and regions. Results: Most adolescents engaged in PA up to 3 days/week (57.1%; 95% CI: 56.9; 57.2). The prevalence of engaging in PA every day decreases over the age from 28.2% at age of 11–12 years (95% CI: 27.4; 29.0) to 21.2% at age of 16–17 years (95% CI: 20.3; 22.0) among boys; and from 19.4% (95% CI: 18.5; 20.2) to 11.1% (95% CI: 10.1; 12.0) among girls. For boys and girls who engaged in PA 5-6 days/week, the prevalence increases from countries with the lowest human development index to countries with the highest. Cambodia (7.3%, 95% CI: 3.8; 10.8), Philippines (7.7%, 95% CI: 5.6; 9.7), Sudan (8.8%, 95% CI: 4.7; 12.9), Timor-Leste (8.9%, 95% CI: 5.5; 12.3), and Afghanistan (10.1%, 95% CI: 6.1; 14.1) were the countries with the lowest prevalence of sufficient PA. Conclusions: National, regional, and worldwide data on the prevalence of physical activity in adolescents highlights the importance of improving the global levels of PA, especially in girls. Identifying the factors causing the age-related decrease in physical activity levels will permit public health entities to define priority actions and policies against physical inactivity.

## 1. Introduction

Physical activity (PA) is an important health behaviour. Among children and adolescents, PA has a significant impact on cardiovascular, bone, and metabolic health, improving fitness, weight status, and sleep [1]. Besides physical health benefits, there is increasing evidence on the cognitive, psychological, and social benefits of PA [2]. Furthermore, PA benefits obtained during adolescence can be transferred into adulthood [1,3]. To achieve these benefits, adolescents are recommended to engage in moderate-to-vigorous intensity PA at least 60 min every day [4]. 

Promoting PA is of importance to public health. Even if PA recommendations are not attained, major improvements in health can be achieved from modest increases in regular PA [5]. Furthermore, some evidence suggests that those who are below the current public health recommendations benefit more from increases in PA than those who are already within the recommendations [1]. Thus, the public health impact of PA and the potential benefits of even small population-wide increases are considerable [1].

Despite all the health benefits of PA, most adolescents worldwide are physically inactive. It is estimated that 77.6% of boys and 84.7% of girls aged 11 to 17 years old are physically inactive [6]. The evidence also suggests that PA declines during adolescent years [6,7]. Updated information on the prevalence and trends of physical inactivity is important to inform national and international authorities and support countries’ public health policies and actions. Nonetheless, previous studies focused on the percentage of adolescents who did not practice enough PA to achieve PA recommendations [6,7,8]. Thus, all adolescents who did not achieve the PA recommendations were grouped in the same group. However, considering in the same group adolescents who do not practice enough PA on any day of the week with adolescents who practice 3 or more days/week for at least 60 min of PA may not be useful for policymakers and public health promoters. Knowledge of PA participation prevalence according to frequency, i.e., times practiced per week, is also crucial as it presents more detailed data and allows for further action and tailored public health strategies in each country. Thus, the aim of this study, using cross-sectional survey data, was to present the worldwide, regional, and national prevalence of PA participation according to its frequency in adolescents aged 11 to 17 years.

## 2. Materials and Methods 

### 2.1. Participants and Procedures

This study is based on cross-sectional surveys of adolescents’ populations from several countries, from all regions worldwide. Data were collected from representative national sample sizes of at least 100 adolescents, from 105 countries. Data were from national and international surveys, namely the Health Behaviour in School-aged Children (HBSC), the Global School-Based Student Health Survey (GSHS), the Youth Risk Behavior Surveillance (YRBS), the Pesquisa Nacional de Saúde do Escolar (PENSE), and the Encuesta Nacional de Salud y Nutrición (ENSNUT). The final database, with data from all surveys, consisted of 558,443 adolescents. As the surveys did not collect data from adolescents within the same age range, only adolescents aged 11 to 17 years were selected to allow comparison between countries. Adolescents with missing data/non-response on PA were removed from the sample. The final sample comprised 520,533 adolescents (251,788 boys, 268,745 girls) from 105 countries and eight regions.

Data collected in surveys were self-reported. Adolescents were asked to report the number of days they were physically active for a total of at least 60 min per day in the last 7 days. Answers were given on an 8-point scale (0 = none to 7 = daily). This is a valid and reliable question assessing adolescents’ PA at an epidemiological level [9,10], and it has been used in several studies [6,11,12,13]. Although the data came from different surveys (HBSC, GSHS, YRBS, PENSE, ENSANUT), it should be noted that in all surveys the same question was used to assess the adolescents’ PA levels. Adolescents were provided with a definition of PA accompanied by examples of some age-relevant activities (e.g., running, cycling, dancing, playing football, basketball).

### 2.2. Data Analysis

For the entire sample, descriptive statistics were calculated (percentages and 95% confidence interval). To estimate the prevalence of PA levels, data were stratified by sex, age (11 to 12 years, 13 to 15 years, 16 to 17 years), eight world regions (1. Central and Eastern Europe, 2. Central Asia, the Middle East, and North Africa, 3. East and southeast, Asia, 4. High-income Western countries, 5. Oceania, 6. Sub-Saharan Africa, 7. South Asia, 8. Latin America and the Caribbean), adapted from the World Bank and previous studies [6,14], human development index (HDI) (low, middle, high, very high), and countries [15]. PA was recoded into 1 to 2 days/week, 3 to 4 days/week, 5 to 6 days/week, and every day. The daily practice of moderate to vigorous PA of at least 60 min was defined as reaching the PA recommendation [4]. Descriptive data are presented as percentages. The prevalence of PA levels was calculated along with a 95% confidence interval (CI). Data analysis was performed using IBM SPSS Statistics version 25.

## 3. Results

Table 1 presents the sample distribution according to age, HDI, and PA levels. Boys and girls presented different figures. About half of the boys (50.1%; 95% CI: 49.8, 50.2) engaged in PA up to 3 days/week and for girls, the prevalence was 63.6% (95% CI: 63.4, 63.8). For every day PA, boys presented a higher prevalence than girls (22.1%; 95% CI: 21.7, 22.4 vs. 12.6%, 95% CI: 12.2, 12.9).

In Table 2, the prevalence of PA levels stratified by age groups is presented. The prevalence of the lowest level of PA (never) increases from 11 to 12 years to 16 to 17 years among boys and girls. Overall, the biggest increase is from 11 to 12 years to 13 to 15 years. From the ages of 13 to 15 to 16 to 17 years, the increase is slight. On the other hand, the prevalence of engaging in PA every day decreases over the age from 28.2% (95% CI: 27.4, 29.0) to 21.2% (95% CI: 20.3, 22.0) among boys, and from 19.4% (95% CI: 18.5, 20.2) to 11.1% (95% CI: 10.1, 12.0) among girls. The prevalence of PA 3 to 4 days/week and 5 to 6 days/week also decreases with age among boys and girls.

The levels of PA according to the HDI is presented in Table 3. The prevalence of never engaging in PA of at least 60 min/day is more pronounced in countries with the lowest HDI. For boys and girls, the prevalence decreases moderately among low HDI countries to high HDI countries. However, there is a very sharp decrease in countries with very high HDI. In boys and girls who engaged in PA 5 to 6 days/week, the prevalence increases from the countries with the lowest HDI to the countries with the highest HDI. For PA every day, boys from countries with very high HDI presented a slightly higher prevalence (24.7%, 95% CI: 24.2, 25.2) compared to those from countries with high, middle and low HDI. For the girls, no clear pattern was observed. The highest prevalence for achieving PA recommended levels were from the countries with the lowest HDI (16.5%, 95% CI: 14.8, 18.2) and highest HDI (14.7%, 95% CI: 14.2, 15.2).

The prevalence of PA levels according to regions is presented in Table 4. Across all regions, girls were less active than boys. For boys and girls, the lowest prevalence of never doing PA was observed in Central and Eastern Europe, and in high-income Western countries. Conversely, South Asian countries presented the highest prevalence. The regions that showed the highest prevalence of never doing PA are the same that showed the highest prevalence for doing PA daily. Among girls, the results were quite different. Girls from South Asia were the ones that had the highest prevalence in achieving PA recommended levels.

Cambodia (7.3%, 95% CI: 3.8, 10.8), Philippines (7.7, 95% CI: 5.6, 9.7), Sudan (8.8, 95% CI: 4.7, 12.9), Timor-Leste (8.9, 95% CI: 5.5, 12.3), and Afghanistan (10.1, 95% CI: 6.1, 14.1) were the countries with the lowest prevalence of sufficient PA. Three of those countries are from East and Southeast Asia (Cambodia, Philippines, and Timor-Leste). From the other regions, the countries with the lowest prevalence of sufficient PA were Italy from the High-income Western countries (10.3%, 95% CI: 7.3, 13.2), Syria from Central Asia, Middle East, and North Africa (10.6%, 95% CI: 7.2, 13.9), Vanuatu from Oceania (11%, 95% CI: 5.4, 16.6), Curaçao from Latin America and the Caribbean (11.7, 95% CI: 7.7, 15.8), Mauritania from Sub-Sharan Africa (12.1%, 95% CI: 7.9, 16.3), and Estonia from Central and Eastern Europe (16.4%, 95% CI: 13.6, 19.3). Countries that presented the highest prevalence of PA every day, achieving the PA recommended levels, were Finland (27.9, 95% CI: 25.7, 30.0), Albania (28.0, 95% CI: 25.6, 30.3), Bulgaria (28.6, 95% CI: 26.2, 31.0), the United States (32.3, 95% CI: 30.9, 33.8), and Bangladesh (48.2, 95% CI: 45.6, 50.8). Considering PA ≥5 days per week, countries that presented the highest prevalence were Iceland (53.7, 95% CI: 52.3, 55.0), the United States (56.7, 95% CI: 55.6, 57.9), Canada (58.0, 95% CI: 56.9, 59.1), Finland (58.7, 95% CI: 57.0, 60.3), and Bangladesh (59.7, 95% CI: 57.4, 62.1). Table A1 shows the physical activity levels by regions and countries (Appendix A).

## 4. Discussion

This study found that the prevalence of adolescents doing sufficient PA was low. Less than 20% of the adolescents engaged in PA every day and almost 20% never engaged in PA. Regardless of the country, region, HDI, or age group, adolescent girls revealed consistently lower PA levels than boys. These results reinforce the urgent need for worldwide international, national, and local agencies to take action to promote PA, especially among girls. Interventions conducted for improving girls’ PA have shown small but significant effects [16]. Successful strategies for changing girls’ PA were found for interventions that were theory-driven, multi-component in design, school-based, and that also offer physical education, which addresses the unique needs of girls and targeting girls only [16,17]. Engagement with young people, such as active listening to their voices, has also been identified as an important way of developing meaningful PA opportunities [18,19]. 

The scaling up of implementing known effective policies and programs as well as social marketing campaigns combined with community-based interventions have been suggested for increasing PA in adolescents, particularly in girls [6]. Overall, recent evidence of PA trend studies indicates that the percentage of physically active adolescent girls has not increased over the years [6,20]. This suggests that programs and policies to promote PA among adolescent girls are having limited success. Nevertheless, in another trend study, an increase in the prevalence of PA among girls has been found aged 11 to 15 years from 10 out of 32 countries [21]. Understanding why these countries succeed in improving girls’ PA levels might be important in helping to define and implement more effective policies and strategies.

Another major finding from our study was that sufficient PA decreases with age among boys and girls, which is in line with previous literature [22]. In our study, a decrease with age seems to be similar in both sexes, i.e., around 7% to 8% from 11 to 12 years to 16 to 17 years. However, the biggest decrease in meeting the PA levels is from 11 to 12 years to 13 to 15 years. Therefore, early adolescence seems to be a critical period for either preventing the PA decline or promoting PA. Furthermore, considering that PA decline starts at ages 6 to 7 [22], that PA may track from childhood to adolescence [23], that childhood is a critical period for developing the basic motor competencies [24], and the fact that in our study 11-year-old boys and girls had already low levels of PA, there is a need for a greater emphasis on promoting PA from an early age (and not only in adolescence).

It was found that the prevalence of never engaging in PA of at least 60 min per day was more pronounced in countries with the lowest HDI and decreased for countries with higher HDI. Additionally, the prevalence of engaging in PA 5 to 6 days/week was higher in countries with the highest HDI. A similar trend was observed among low- and middle-income countries, where increased HDI values were associated with decreased levels of physical inactivity [25]. In contrast, the analyses based on the 2018 Activity Report Card for children and youth showed higher PA levels in the low and medium HDI countries [8]. Additionally, the prevalence of insufficient PA in analysis with adults was more than twice as high in high-income countries as in low-income countries [26]. Finally, less economically developed countries had the lowest prevalence of physical inactivity, while physical inactivity was more prevalent among the most developed economic countries [7]. The different findings between studies can be in part explained based on compositional differences in the study sample and PA measurement method. For instance, in Activity Report Cards the PA assessment used indicators that included 5 behaviors (overall PA, organized sports participation, active play, active transportation, sedentary behavior) [8], while for the present study a single-item indicator of total PA was used. Furthermore, the fact that PA also varies across countries suggests that the factors influencing PA lie mostly at the national community level [26]. Nevertheless, these conflicting results highlight an area for future research to better understand the factors affecting the relationship between PA and HDI.

The results of this investigation have several implications for national and international public health policies. Overall, this investigation reinforces the World Health Organization’s recommendation for countries to develop or update national policy and implementation plans on PA [27] as findings suggest an urgent need to improve PA levels among adolescents. One critical aspect is the necessity of allocating the necessary political priority and resources to enable the implementation of PA policies [6].

For every single country, age and sex were factors associated with PA participation. In this regard, national public health policies aiming to promote PA should also focus on reducing the gap between sexes and age groups. Thus, effectively promoting PA among adolescents requires identifying, understanding, and intervening on the causes and inequities [6]. Furthermore, because of the low levels of PA, countries would benefit and should invest in the promotion of PA in all its forms (including recreational PA, sports participation, physical education, and active travel) and in all sectors. This multisectoral approach, leading to the involvement of health authorities, schools, sports clubs and federations, regional planners, and political leaders on PA promoting strategies, is considered effective and commended in the WHO Global action plan on PA 2018-2030 [28].

Several limitations of this study are important to mention. Data were collected at the school setting resulting in the exclusion of adolescents that do not attend schools. In addition, data were from adolescents aged 11 to 17 years. However, data from the HBSC survey were collected among those who attended the grades 6, 8, and 10. Thus, adolescents from the HBSC survey were mostly aged 11, 13, and 15 years, whereas the GSHS focuses on adolescents aged 13 to 17 years. Furthermore, data were self-reported and subject to bias. Despite large samples used in each national or regional survey, self-report was the most feasible methodology and is still the backbone of surveillance studies [29].

## 5. Conclusions

Monitoring PA levels in adolescents allows the identification of specific needs and inequities within and between countries, and consequently promotes informed decisions and actions. Findings show that worldwide PA levels among adolescents are low, as 14.9% of boys and 21.2% of girls report to never practice PA, and that the situations get worse with aging. Identifying and understanding the main factors related to the insufficient and decreased PA levels, will permit international and national public health entities to define priority actions and policies against physical inactivity. Reducing the gender gap, specifically improving girls’ PA levels should be considered a priority in public health-related actions. This process, initiated with national or regional surveillance of PA, ultimately aims to improve PA levels and promotes healthy lifestyles among adolescents as well as carrying them forward into adulthood.

## Figures and Tables

**Table 1:** Participants’ characteristics.

	Total (n = 520,533)		Boys (n = 25,1788)		Girls (n = 268,745)	
	n	% (95% CI)	n	% (95% CI)	n	% (95% CI)
**Age**						
11 years	55,782	10.7 (10.5, 11.0)	27,153	10.8 (10.4, 11.2)	28,629	10.7 (10.3, 11.0)
12 years	25,609	4.9 (4.7, 5.2)	12,246	4.9 (4.5, 5.2)	13,363	5.0 (4.6, 5.3)
13 years	116,031	22.3 (22.1, 22.5)	54,536	21.7 (21.3, 22.0)	61,495	22.9 (22.6, 23.2)
14 years	116,894	22.5 (22.2, 22.7)	55,184	21.9 (21.6, 22.3)	61,710	23.0 (22.6, 23.3)
15 years	131,520	25.3 (25.0, 25.5)	64,961	25.8 (25.5, 26.1)	66,559	24.8 (24.4, 25.1)
16 years	53,582	10.3 (10.0, 10.6)	27,251	10.8 (10.5, 11.2)	26,331	9.8 (9.4, 10.2)
17 years	21,115	4.1 (3.8, 4.3)	10,457	4.2 (3.8, 4.5)	10,658	4.0 (3.6, 4.3)
**HDI**						
Very high	225,408	43.3 (43.1, 43.5)	110,258	43.8 (43.5, 44.1)	115,150	42.8 (42.6, 43.1)
High	185,430	35.6 (35.4, 35.8)	89,604	35.6 (35.3, 35.9)	95,826	35.7 (35.4, 36.0)
Middle	87,088	16.7 (16.5, 17.0)	40,249	16.0 (15.6, 16.3)	46,839	17.4 (17.1, 17.8)
Low	22,607	4.3 (4.1, 4.6)	11,677	4.6 (4.3, 5.0)	10,930	4.1 (3.7, 4.4)
**PA**						
Never	94,279	18.1 (17.9, 18.4)	37,395	14.9 (14.5, 15.2)	56,884	21.2 (20.8, 21.5)
1 days/week	69,607	13.4 (13.1, 13.6)	29,089	11.6 (11.2, 11.9)	40,518	15.1 (14.7, 15.4)
2 days/week	68,091	13.1 (12.8, 13.3)	29,280	11.6 (11.3, 12.0)	38,811	14.4 (14.1, 14.8)
3 days/week	64,870	12.5 (12.2, 12.7)	30,111	12.0 (11.6, 12.3)	34,759	12.9 (12.6, 13.3)
4 days/week	50,501	9.7 (9.4, 10.0)	25,403	10.1 (9.7, 10.5)	25,098	9.3 (9.0, 9.7)
5 days/week	51,611	9.9 (9.7, 10.2)	27,098	10.8 (10.4, 11.1)	24,513	9.1 (8.8, 9.5)
6 days/week	32,193	6.2 (5.9, 6.4)	17,855	7.1 (6.7, 7.5)	14,338	5.3 (5.0, 5.7)
Every day	89,381	17.2 (16.9, 17.4)	55,557	22.1 (21.7, 22.4)	33,824	12.6 (12.2, 12.9)

Abbreviation: HDI, Human development index; PA, physical activity.

**Table 2:** Physical activity levels according to age, stratified by sex.

	Age		
	11–12 Years	13–15 Years	16–17 Years
**Boys**	% (95% CI)	% (95% CI)	% (95% CI)
Never	6.7 (5.8, 7.7)	16.0 (15.6, 16.5)	17.9 (17.0, 18.9)
1–2 days/week	16.7 (15.8, 17.6)	23.6 (23.2, 24.0)	28.0 (27.1, 28.9)
3–4 days/week	24.9 (24.1, 25.8)	22.0 (21.6, 22.4)	19.1 (18.2, 20.0)
5–6 days/week	23.4 (22.6, 24.3)	17.5 (17.0, 17.9)	13.8 (12.9, 14.7)
Every day	28.2 (27.4, 29.0)	20.9 (20.5, 21.3)	21.2 (20.3, 22.0)
**Girls**	% (95% CI)	% (95% CI)	% (95% CI)
Never	8.4 (7.5, 9.4)	23.1 (22.7, 23.5)	25.6 (24.7, 26.5)
1–2 days/week	21.9 (21.0, 22.7)	30.0 (29.6, 30.4)	35.7 (34.8, 36.5)
3–4 days/week	28.3 (27.5, 29.1)	21.9 (21.5, 22.3)	17.3 (16.4, 18.3)
5–6 days/week	22.1 (21.2, 22.9)	13.6 (13.2, 14.0)	10.4 (9.4, 11.3)
Every day	19.4 (18.5, 20.2)	11.4 (11.0, 11.8)	11.1 (10.1, 12.0)

**Table 3:** Physical activity levels according to the human development index, stratified by sex.

	Human Development Index			
	Low	Middle	High	Very High
**Boys**	% (95% CI)	% (95% CI)	% (95% CI)	% (95% CI)
Never	30.2 (28.7, 31.7)	25.3 (24.5, 26.2)	19.1 (18.6, 19.7)	5.9 (5.3, 6.5)
1–2 days/week	32.1 (30.7, 33.6)	31.9 (31.1, 32.7)	24.2 (23.7, 24.8)	18.2 (17.7, 18.7)
3–4 days/week	12.0 (10.3, 13.7)	14.8 (13.9, 15.7)	20.7 (20.1, 21.3)	26.9 (26.4, 27.4)
5–6 days/week	6.8 (5.0, 8.6)	9.6 (8.6, 10.5)	15.1 (14.5, 15.7)	24.3 (23.8, 24.8)
Every day	18.9 (17.3, 20.5)	18.4 (17.5, 19.3)	20.8 (20.2, 21.4)	24.7 (24.2, 25.2)
**Girls**	% (95% CI)	% (95% CI)	% (95% CI)	% (95% CI)
Never	36.4 (34.9, 37.9)	30.2 (29.5, 31.0)	31.0 (30.5, 31.6)	7.8 (7.2, 8.4)
1–2 days/week	30.9 (29.3, 32.4)	35.6 (34.9, 36.3)	29.9 (29.3, 30.4)	26.6 (26.1, 27.1)
3–4 days/week	9.8 (8.0, 11.6)	14.0 (13.1, 14.8)	18.3 (17.7, 18.9)	30.1 (29.6, 30.6)
5–6 days/week	6.4 (4.6, 8.2)	8.3 (7.5, 9.2)	10.8 (10.2, 11.4)	20.7 (20.2, 21.3)
Every day	16.5 (14.8, 18.2)	11.8 (11.0, 12.7)	10.0 (9.4, 10.6)	14.7 (14.2, 15.2)

**Table 4:** Physical activity levels according to regions, stratified by sex.

	Region							
	Central and Eastern Europe	Central Asia, Middle East, and North Africa	East and Southeast Asia	High-Income Western Countries	Oceania	Sub-Saharan Africa	South Asia	Latin America and Caribbean
**Boys**	% (95% CI)	% (95% CI)	% (95% CI)	% (95% CI)	% (95% CI)	% (95% CI)	% (95% CI)	% (95% CI)
Never	3.3 (2.4, 4.3)	19.0 (17.8, 20.3)	23.5 (22.4, 24.6)	3.5 (2.7, 4.2)	26.4 (24.2, 28.6)	27.5 (25.7, 29.3)	35.1 (33.1, 37.2)	22.3 (21.7, 22.9)
1–2 days/week	15.0 (14.1, 15.9)	32.4 (31.3, 33.6)	34.0 (33.0, 35.0)	16.2 (15.5, 16.9)	31.0 (28.8, 33.2)	30.9 (29.2, 32.6)	28.6 (26.4, 30.7)	25.4 (24.9, 26.0)
3–4 days/week	27.2 (26.3, 28.0)	18.1 (16.8, 19.4)	16.0 (14.9, 17.1)	28.8 (28.1, 29.4)	13.9 (11.5, 16.4)	15.1 (13.2, 17.0)	9.8 (7.4, 12.3)	19.2 (18.6, 19.8)
5–6 days/week	25.9 (25.1, 26.7)	8.7 (7.3, 10.0)	8.7 (7.5, 9.9)	26.8 (26.2, 27.5)	12.9 (10.5, 15.4)	8.2 (6.2, 10.2)	6.3 (3.8, 8.8)	14.0 (13.3, 14.6)
Every day	28.6 (27.8, 29.4)	21.7 (20.5, 23.0)	17.8 (16.6, 18.9)	24.7 (24.0, 25.4)	15.7 (13.3, 18.1)	18.3 (16.4, 20.1)	20.2 (17.9, 22.5)	19.1 (18.5, 19.7)
**Girls**	% (95% CI)	% (95% CI)	% (95% CI)	% (95% CI)	% (95% CI)	% (95% CI)	% (95% CI)	% (95% CI)
Never	4.5 (3.6, 5.4)	26.3 (25.2, 27.5)	26.8 (25.8, 27.8)	4.4 (3.6, 5.1)	30.0 (28.0, 32.0)	34.9 (33.3, 36.4)	37.0 (34.7, 39.4)	35.6 (35.1, 36.2)
1–2 days/week	21.7 (20.9, 22.6)	39.2 (38.2, 40.3)	44.1 (43.2, 45.0)	24.2 (23.6, 24.9)	31.3 (29.4, 33.3)	33.5 (31.9, 35.1)	22.6 (20.0, 25.2)	29.9 (29.3, 30.4)
3–4 days/week	31.9 (31.1, 32.7)	15.2 (13.9, 16.4)	15.3 (14.2, 16.4)	33.6 (33.0, 34.2)	13.8 (11.6, 16.0)	13.1 (11.3, 14.9)	8.0 (5.1, 10.8)	15.5 (14.9, 16.1)
5–6 days/week	23.4 (22.6, 24.2)	6.8 (5.6, 8.1)	6.0 (4.8, 7.1)	23.4 (22.7, 24.0)	11.5 (9.3, 13.7)	6.0 (4.1, 7.9)	8.4 (5.5, 11.3)	9.6 (8.9, 10.2)
Every day	18.5 (17.6, 19.3)	12.5 (11.2, 13.7)	7.9 (6.8, 9.0)	14.4 (13.7, 15.1)	13.4 (11.2, 15.6)	12.5 (10.7, 14.4)	24.0 (21.4, 26.6)	9.4 (8.7, 10.0)

## Appendix A

**Table A1:** Physical activity levels by region and countries.

Region	Country	Physical Activity (%)				
		Never	1–2 Days/Week	3–4 Days/Week	5–6 Days/Week	Every Day
Central and eastern Europe	Albania	3.1	21.0	30.2	17.8	28.0
	Bulgaria	4.5	18.4	26.5	22.0	28.6
	Canada	3.7	12.7	25.6	33.0	25.0
	Croatia	2.6	17.7	27.5	26.5	25.6
	Czech Republic	2.5	17.7	32.9	25.7	21.3
	Estonia	3.6	21.1	35.1	23.8	16.4
	Greenland	10.7	31.7	22.7	16.8	18.0
	Hungary	5.1	18.8	29.0	24.5	22.6
	Latvia	3.1	20.8	33.6	24.0	18.4
	Macedonia	2.7	14.7	31.1	24.6	26.9
	Moldova	3.7	23.8	24.6	21.8	26.0
	Poland	4.3	16.3	27.9	27.6	24.0
	Romania	9.6	26.8	23.7	18.7	21.3
	Russia	5.0	19.8	38.6	18.7	17.9
	Slovakia	3.2	17.8	29.6	24.1	25.3
	Slovenia	3.1	20.1	32.4	25.9	18.5
	Ukraine	4.5	16.5	32.0	20.8	26.3
Central Asia, Middle East, and north Africa	Algeria	11.4	53.7	14.0	5.8	15.1
	Armenia	4.9	19.9	34.6	17.8	22.9
	Egypt	30.4	40.9	10.7	3.6	14.5
	Iraq	37.1	31.4	11.9	5.0	14.6
	Kuwait	22.6	32.8	19.5	9.6	15.5
	Lebanon	16.3	29.3	18.1	11.7	24.6
	Mongolia	13.9	30.4	19.5	11.0	25.2
	Morocco	22.0	49.0	11.0	4.8	13.1
	Oman	27.9	36.8	19.5	4.1	11.7
	Palestinian territory	33.8	33.3	11.4	5.8	15.7
	Syria	35.6	39.2	10.7	3.9	10.6
	United Arab Emirates	19.0	32.8	20.3	9.9	18.0
	Yemen	38.8	35.2	9.7	4.4	11.9
East and southeast Asia	Brunei Darussalam	16.4	38.9	23.5	9.2	12.1
	Cambodia	38.2	42.8	8.3	3.3	7.3
	Laos	24.3	43.5	11.5	5.5	15.2
	Malasya	17.9	39.0	19.1	9.1	14.9
	Philippines	44.3	34.6	8.8	4.6	7.7
	Thailand	23.7	38.7	17.8	8.1	11.9
	Timor-Leste	30.2	49.6	8.2	3.2	8.9
	Viet Nam	27.6	38.8	14.2	6.2	13.1
High-income western countries	Austria	2.4	20.1	32.9	25.0	19.4
	Belgium	3.6	23.3	34.1	22.5	16.6
	Denmark	4.9	23.1	35.6	23.2	13.1
	England	2.4	17.1	33.5	28.5	18.5
	Finland	1.8	11.3	28.2	30.8	27.9
	France	4.8	27.2	35.0	20.1	12.9
	Germany	3.7	22.7	37.4	20.8	15.4
	Greece	6.5	23.2	34.3	22.6	13.4
	Iceland	4.6	13.9	27.9	31.2	22.4
	Ireland	3.4	14.3	29.6	29.1	23.6
	Israel	16.3	34.9	24.3	12.1	12.4
	Italy	8.3	28.7	33.8	18.9	10.3
	Luxembourg	2.8	19.6	30.4	24.4	22.8
	Malta	7.3	24.3	28.2	22.3	17.9
	Netherlands	3.2	15.5	31.8	31.3	18.3
	Norway	3.0	16.6	33.7	27.9	18.9
	Portugal	3.6	28.7	32.9	19.0	15.8
	Scotland	3.1	16.7	34.0	28.7	17.5
	Spain	3.0	15.0	30.2	25.9	25.9
	Sweden	4.5	19.2	34.4	27.8	14.1
	Switzerland	2.1	18.4	36.0	29.1	14.4
	United States		22.2	21.1	24.4	32.3
	Wales	4.0	20.6	35.5	23.5	16.3
Oceania	Fiji	26.0	27.3	14.7	13.5	18.5
	Kiribati	28.0	32.9	12.5	8.8	17.8
	Nauru	49.2	19.3	11.0	7.9	12.6
	Niue	27.7	21.9	19.0	19.0	12.4
	Samoa	28.3	32.5	18.3	9.0	11.8
	Solomon Islands	18.9	38.9	15.4	10.5	16.4
	Tokelau	33.1	17.4	11.6	14.0	24.0
	Tonga	32.4	31.1	11.6	11.1	13.8
	Tuvalu	47.0	26.7	9.4	5.4	11.6
	Vanuatu	17.7	29.2	8.1	34.0	11.0
	Wallis and Futuna	20.7	37.3	19.5	9.1	13.3
Sub-Sharan Africa	Ghana	32.7	32.6	14.8	6.9	13.0
	Mauritania	41.1	34.3	8.0	4.5	12.1
	Mauritius	18.0	31.3	19.1	12.4	19.2
	Mozambique	21.4	45.4	13.8	6.0	13.4
	Namibia	41.4	26.3	11.8	6.5	14.1
	Seychelles	31.3	28.1	16.5	7.2	16.9
	Sudan	33.0	40.5	14.2	3.5	8.8
	Tanzania	27.3	30.5	14.0	8.1	20.2
South Asia	Afghanistan	30.1	36.5	14.7	8.6	10.1
	Bangladesh	23.1	9.9	7.3	11.6	48.2
	Pakistan	45.7	30.8	7.7	4.1	11.7
Latin America and Caribbean	Antiqua and Barbuda	30.1	21.1	16.2	9.8	22.7
	Argentina	15.1	35.0	20.2	11.5	18.2
	Bahamas	31.9	28.8	15.8	8.9	14.6
	Barbados	28.1	27.6	16.7	9.4	18.3
	Belize	32.4	21.8	15.4	9.5	20.9
	Bolivia	22.3	38.9	15.9	9.4	13.5
	Brasil	34.2	24.1	16.8	12.9	12.0
	British Virgin Islands	31.3	26.9	16.2	7.7	18.0
	Chile	16.5	32.0	23.9	12.4	15.2
	Costa Rica	18.0	35.1	19.2	9.6	18.1
	Curaçao	32.3	31.9	16.5	7.6	11.7
	Dominica	35.1	28.7	13.3	6.8	16.1
	El Salvador	28.6	35.4	13.7	8.5	13.8
	Guatemala	26.9	35.9	15.9	7.2	14.0
	Guyana	39.2	26.2	11.8	7.2	15.6
	Honduras	28.9	37.3	12.3	5.8	15.7
	Mexico	17.5	38.5	16.8	9.3	17.8
	Peru	17.8	37.7	19.3	9.8	15.4
	Saint Kitts and Nevis	32.7	26.9	15.3	7.2	18.0
	Suriname	26.0	30.8	16.6	7.4	19.2
	Trinidad and Tobago	29.8	26.4	15.6	8.3	19.9
	Uruguay	20.7	31.0	20.7	12.4	15.2
